# A past Haff disease outbreak associated with eating freshwater pomfret in South China

**DOI:** 10.1186/1471-2458-13-447

**Published:** 2013-05-06

**Authors:** Xi Huang, Yipeng Li, Qiong Huang, Junhua Liang, Chunsui Liang, Bifeng Chen, Lingling Lu, Xiaoling Deng, Zihui Chen, Yonghui Zhang, Yongning Wu, Bing Shao

**Affiliations:** 1 Guangdong Provincial Centre for Disease Control and Prevention, Guangzhou, China; 2 School of Public Health, Sun Yat-sen University, Guangzhou, China; 3 Jiangmen Prefectural Centre for Disease Control and Prevention, Jiangmen, China; 4 Guangdong Provincial Institute of Public Health, Guangzhou, China; 5 National Centre for Food Safety Risk Assessment, Beijing, China; 6 Beijing Centre for Disease Control and Prevention, Beijing, China

## Abstract

**Background:**

Haff disease is unexplained rhabdomyolysis caused by consumption of fishery products in the previous 24 h. It was first identified in Europe in 1924 but the condition is extremely rare in China. Here we describe a past outbreak of acute food borne muscle poisoning that occurred in Guangdong Province (South China) in 2009.

**Methods:**

The first full outbreak of Haff disease reported in Jiangsu Province (East China) in 2010, indicated that the incidence of the disease may be increasing in China. We, therefore first retrospectively reviewed epidemiologic, trace-back, environmental studies, and laboratory analyses, including oral toxicity testing to ascertain risk and chemical analysis to identify toxin(s), from the 2009 Guangdong outbreak. Then we compared data from the 2009 outbreak with data from all other Haff disease outbreaks that were available.

**Results:**

Clinical symptoms and laboratory findings indicated that the 2009 Guangdong outbreak disease was consistent with rhabdomyolysis. Epidemiologic, trace-back, environmental studies and laboratory analyses implied that the disease was caused by freshwater Pomfrets consumed prior to the onset of symptoms. We also identified common factors between the 2009 Guangdong outbreak and previous Haff disease outbreaks reported around the world, while as with other similar outbreaks, the exact etiological factor(s) of the disease remains unknown.

**Conclusions:**

The 2009 Guangdong outbreak of ‘muscle poisoning’ was retrospectively identified as an outbreak of Haff disease. This comprised the highest number of cases reported in China thus far. Food borne diseases emerging in this unusual form and the irregular pattern of outbreaks present an ongoing public health risk, highlighting the need for improved surveillance and diagnostic methodology.

## Background

Rhabdomyolysis, is characterized by disintegration of striated muscle fibers resulting in intracellular contents being excreted into the circulatory system. It is not an uncommon occurrence but can be potentially life-threatening, especially if the intracellular contents are filtered by the glomerulus, as myoglobin obstructs the renal tubules causing acute renal failure [1-3]*.* Physical symptoms of rhabdomyolysis include profound muscular pain and weakness, swelling, stiffness, cramps, and sometimes tea-colored urine. The etiology of rhabdomyolysis is diverse being classified as traumatic, exercise-induced, toxicological, environmental, metabolic, infectious, immunologic, pharmacotherapeutic, and inherited [1]. A 5-fold or greater increase in serum creatine kinase (CK), in the absence of heart or brain diseases, and a CK muscle/brain (MB) fraction < 5%, are widely accepted diagnostic criteria [2,4].

Haff disease, the occurrence of unexplained rhabdomyolysis in a person who has consumed cooked fishery products within the previous 24 h, is a relatively uncommon condition [5]. It was first identified in 1924 near the Haff shores along the Baltic Coast in East Prussia. Additional cases and outbreaks were subsequently described in the same region, and also in several other countries [6]. Haff disease has seldom been reported in China or Asia, and outbreaks have been even more uncommon. On 27 October 2009, there was an outbreak of unexplained myalgia after consumption of freshwater Pomfret *(Colossoma brachypomum)*, in a town in Lianzhou County, Guangdong Province, South China. The outbreak was described as an incident of acute muscle poisoning. Between July and August, 2010, a crayfish-related Haff disease outbreak involving 23 cases, was reported in Nanjing (Provincial Capital city), Jiangsu Province, in East China [7]. This second outbreak focused our attention towards the risks of Haff disease in China. It also prompted us to review Haff disease more thoroughly. Part of this research involved a retrospective review of the 2009 Guangdong outbreak.

## Methods

### Outbreak identification

In the early morning of 27 October, 2009, six people presenting with unexplained generalized myalgia, weakness and fatigue, were admitted to hospital in a small town named Jiubei, Lianzhou County, Qingyuan Prefectural City, Guangdong Province, China. Each of them reported recent consumption of locally purchased freshwater Pomfrets. The symptoms became more severe and the patients were transferred from the town hospital to a county hospital. By 10.00 h on 27 October, 35 other patients exposed to the same food were identified.

The outbreak was reported from hospital to the Centres for Disease Control and Prevention (CDCs) at county, prefectural and provincial level in Guangdong. Investigations were expedited without delay.

### Epidemiologic studies

Based on preliminary information gained from the initial cases, the outbreak case was defined by the presence of at least one of the following symptoms since 26 October 2009: muscle pain, weakness, nausea, neck or waist or back ache, or limb weakness. Active surveillance for similar patients in local hospitals, and enquiries among residents were used to find additional cases. All cases were interviewed and demographic and clinical information, food-related and personal risk factors were documented [8]. Physicians dealing with patients were interviewed, and the medical records and clinical laboratory values were reviewed and analyzed. Descriptive epidemiologic, clinical and laboratory characters were compared between genders.

For hypotheses generation, the patients were further interviewed using a uniform detailed questionnaire which provided information on consumption of different food items, focusing on the freshwater Pomfrets that had been mentioned by all patients.

### Trace-back and environmental investigations

By interviewing patients, local residents, fish sellers and fisherman, a trace-back investigation of the distribution pathway for the implicated food item was conducted. Environmental investigations along the distribution chain were performed to determine the possible source of contamination. Food (fish) and environmental (water and soil) samples were collected and sent for laboratory analysis.

### Animal tests

Animal tests and chemical analysis were used to ascertain risk and etiological factor(s) during outbreak investigations. Suspected Pomfrets were sampled from the reservoir where they were caught, and from patients’ leftovers. Bighead carp cultivated from the same reservoir and Pomfrets obtained from another aquaculture site were collected as controls. Aquaculture water (surface and bottom water and water from the outlet of a sewage drain) was sampled and compared with control water samples from other sites.

Fish and water samples were subjected to acute oral toxicity tests for toxins, following method and procedures of Chinese National Food Safety Standard ‘Procedures for toxicological assessment of food’ (GB 15193.1-2003), and using ‘Maximal Tolerate Dose’ (MTD). Imitating the usual cooking process, fish samples were steamed at 100°C for 10 to 15 min till well done, and the muscle parts were minced into a meat paste. Water samples were concentrated 10 times (at 60°C and at 76 mmHg pressure). The prepared samples were administered to SPF BALB/c mice via oral gavage at the MTD of 0.3 mL/10 g every 4 h. Each mouse was subjected to three doses. The behavior and survival of the treated mice were observed, and gross anatomy was further done on, if any, dead mouse. The 10 groups of 20 SPF BALB/c mice (half male and half female) used for these experiments were obtained from Guangdong Provincial Medical Experimental Animal Centre (Guangzhou, China). The mice were housed and treated according to the guidelines for mouse care from Guangdong Provincial Centre for Disease Control and Prevention (Guangdong Provincial CDC) Animal Care and Use Committee.

### Chemical analyses

Fish, water and soil samples were tested for microcystin (LR, RR, YR, LF), paralytic shellfish poison (PSP), ractopamine, fluoride (F^-^), chloride (CL^-^), nitrate (NO_3_^-^), nitrite (NO_2_^-^), sulphate (SO_4_^2-^), gelsemine, histamine, and 27 heavy metals including: beryllium (Be), sodium (Na), magnesium (Mg), aluminum (Al), potassium (K), calcium (Ca), vanadium (V), chromium (Cr), manganese (Mn), iron (Fe), cobalt (Co), nickel (Ni), copper (Cu), zinc (Zn), arsenic (As), selenium (Se), molybdenum (Mo), silver (Ag), cadmium (Cd), tin (Sn), antimony (Sb), barium (Ba), thallium (Tl), lead (Pb), thorium (Th), uranium (U), by using chemical analyses technologies and methods including Liquid Chromatography-Mass Spectrometry (LC-MS), Iron Chromatography (IC), Gas chromatography–mass spectrometry (GC-MS), colorimetric method, Inductively Coupled Plasma Mass Spectrometry (ICP-MS) and Atomic Fluorescence Spectrometry (AFS).

### Identification of specific risk factors

Based on data from other studies, we carried out a retrospective cohort analysis using freshwater Pomfret consumption as a risk factor single variable. The analysis involved 3857 residents of Jiubei (patients and healthy individuals) who had been staying in town and who had a limited period of exposure. The residents were interviewed to ascertain if they had consumed locally sold freshwater Pomfret. Data from 24 patients who had consumed Pomfret were used to determine dose–response relationships.

The study was approved by the Ethics Committee of Guangdong Provincial CDC. Oral informed consent to participate in the study was obtained from all study participants (including patients, physicians and local residents enrolled in our studies).

### Statistical analyses

Statistical analysis was conducted using MS Excel, and SPSS version 13.0 statistical software. Between-group comparisons were analyzed using Fisher’s Exact tests and Pearson chi-square tests, and the Mann–Whitney *U* test was used to analyze gender distributions. Curve estimation regression was used to determine dose–response relationships. Values of *P* < 0.05 were considered statistically significant.

## Results

### Epidemiology characteristics

**Figure 1:**
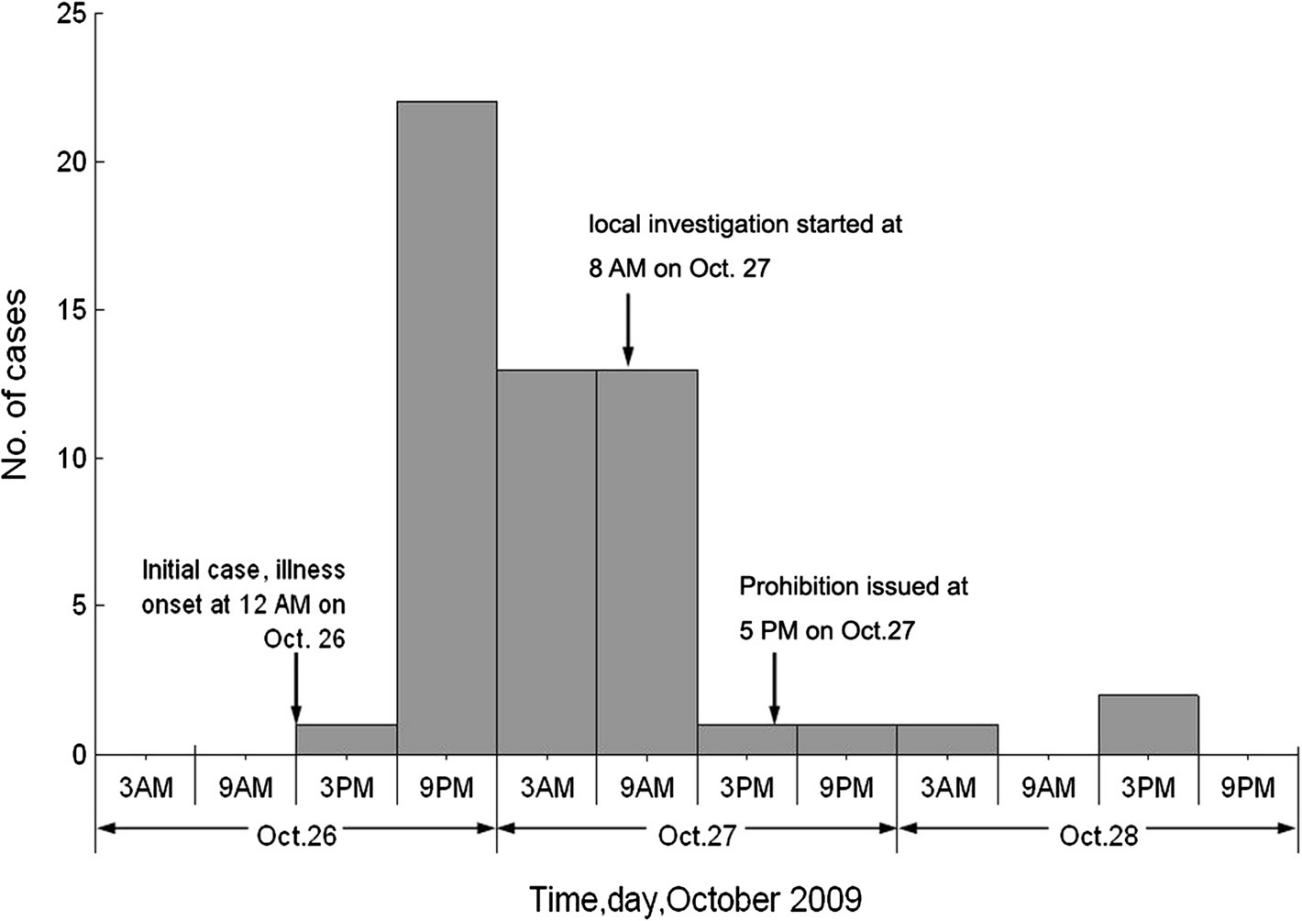
Epidemiologic curve of the outbreak occurred in town of Lianzhou, Guangdong, South China, 2009.

A total of 54 case-patients were identified, among whom 44 were hospitalized. The median length of hospital stay was 3.2 days (range: 1 to 13 days). Only one case was classified as being severe and there were no deaths. The disease onset occurred between 19.00 h on 26 October and 11.00 h on 31 October 2009. The incidence of new cases appeared to peak on the evening of 26 October (Figure 1). The majority of cases were residents of Lianzhou County, and were concentrated in Jiubei town. There were four cases from Liannan, a neighboring county of Lianzhou. Among case-patients, approximately two thirds of the cases were male (male cases: 36/54, 66.7%; female cases: 18/54, 33.3%). Fifty two of the 54 patients were adults (i.e. >18 years of age). The median age of the total population was 43 years (range: 4 to 74 years), and the median age is higher for female case patients than for male cases patients (43 vs. 42 years, range 22 to 74 years vs. 4 to 71 years, a nonsignificant difference, *P* = 0.291) (Table 1). All patients recovered without sequelae and all had been discharged by 9 November 2009. No similar symptoms were reported among these patients afterwards.

**Table 1:** Demographic and clinical characteristics and initial laboratory test results from patients in town of Lianzhou presenting with unexplained myalgia in 2009^*^

**Variable**	**Total (n = 54)**	**Male (n = 36)**	**Female (n = 18)**	P-value*
**Age, years**				0.291
Mean	42	41	45	
Median	43	42	43	
Range	4-74	4-71	22-74	
**Clinical characteristics, n(%)**				
Weakness	45 (83.3)	30 (83.3)	15 (83.3)	1.000
Myalgia	41 (75.9)	28 (77.8)	13 (72.2)	0.740
Nausea	34 (63.0)	23 (63.9)	11 (61.1)	0.842
Abdominal pain	34 (63.0)	24 (66.7)	10 (55.6)	0.425
**Laboratory test values, mean ± SD**				
**Serological test**				
α-HBDH, U/L	357.9 ± 309.6	394.1 ± 342.4	245.7 ± 129.1	0.268
CK, U/L	2812.3 ± 3350.8	3239.5 ± 3674.8	1488.1 ± 1520.0	0.213
AST, U/L	166.7 ± 227.9	193.8 ± 255.4	82.6 ± 55.6	0.514
CK-MB, U/L	99.2 ± 127.0	114.5 ± 141.0	51.9 ± 45.9	0.208
CK-MB Fraction,%	6.0 ± 5.4	5.8 ± 5.1	6.8 ± 6.2	0.632
LDH,U/L	428.4 ± 362.0	473.7 ± 400.2	288.1 ± 137.9	0.302
TBA, μmol/L	25.5 ± 7.00	23.8 ± 6.9	30.6 ± 4.1	0.006
ALT, U/L	64.1 ± 65.8	67.2 ± 72.5	54.6 ± 39.3	0.927
**Renal function test**				
Cr, μmol/L	74.9 ± 15.3	77.0 ± 15.1	67.5 ± 14.9	0.133
BUN, mmol/L	5.3 ± 1.5	5.5 ± 1.3	4.4 ± 1.6	0.048
**Urinalysis**				
SG	1.02 ± 0.007	1.021 ± 0.007	1.020 ± 0.008	0.830

^*^: Data of demographic and clinical characteristics are for all patients identified; data of laboratory test values are for patients who presented to the county level hospital and were taken blood samples for tests.

*: Fisher’s Exact Test and Pearson Chi-Square Test were used for proportions, the Mann–Whitney *U* test was used for gender distribution.

Seven family clusters involving 20 cases were identified among case-patients. A cluster of 15 adult male patients with a median age of 36 years (range: 19 to 62 years) was identified at a construction site. Within this subgroup, 17 construction workers had consumed Pomfrets together at supper on 26 October. An hour later, one member of the group developed waist and back ache, followed by abdominal pain, dry mouth and weakness. His 14 workmates each developed similar symptoms soon after. The attack rate in this subgroup was 88.2% (15/17) and the median incubation period was 5.5 h (range: 1 to 13 h). All 15 workers had been admitted to hospital by 11.00 h on 27 October 2009.

### Clinical characteristics

As shown in Table 1, the most common presenting symptoms were weakness (45 patients, 83.3%), myalgia (41patients, 75.9%), nausea (34 patients, 63.0%) and abdominal pain (34 patients, 63.0%). Other symptoms included dry mouth (25 patients, 46.3%), vomiting (25 patients, 46.3%), diarrhea (18 patients, 33.3%), dizziness (18 patients, 33.3%) and headache (12 patients, 22.2%).

In some cases blood samples were collected at the time of first presentation. Analysis indicated that levels of serum CK, α-hydroxy butyrate dehydrogenase (α-HBDH), aspartate aminotransferase (AST), CK-MB, lactic dehydrogenase (LDH), total bile acid (TBA), and alanine aminotransferase (ALT), were all elevated. In addition, the majority of patients (82.9%) presented with an MB fraction < 5% (Table 2).

**Table 2:** Initial laboratory test values for patients§ in towns of Lianzhou with unexplained myalgia in 2009

**Variable**	**No.**^**†**^	**Mean**	**Median**	**Minimum**	**Maximum**	**Normal range**	**Percentage abnormal**^**‡**^**%**
**Serological test**							
α-HBDH-U/L	37	366.8	191.0	109.0	1223.0	74-140	89.2
CK-U/L	41	2812.3	1302.0	92.0	11696.0	25-200	85.4
AST-U/L	38	175.3	74.8	19.5	1008.4	5-35	76.3
CK-MB-U/L	41	99.2	44.1	12.9	500.1	0-28	65.9
CK-MB fraction%	41	6.0	4.2	0.2	20.6	< 5%	17.1
LDH-U/L	37	447.8	290.0	132.0	1504.0	80-285	61.3
TBA-μmol/L	33	24.9	25.0	8.0	34.8	1.79-7.14	93.9
ALT-U/L	36	63.8	42.9	9.2	320.4	5-40	55.6
**Renal function test**							
Cr, μmol/L	36	74.7	74	39	104	50-120	5.6
BUN, mmol/L	36	5.27	5.5	2.5	8.7	1.79-7.14	8.3
**Urinalysis**							
SG	30	1.021	1.020	1.005	1.030	1.005-1.025	16.7

^§^: Only patients with blood test results were analyzed.

^†^: No. of patients with available serological testing data.

^‡^:No. of patients with abnormalities in serological testing results/No. of patients with available serological testing data.

Available results of renal function test showed creatinine (Cr) levels of some patients remained normal, ranging from 39 μmol/L to 104 μmol/L (reference range: 50–120 μmol/L), and only four patients presented slightly elevated blood urine nitrogen (BUN).

Routine hematology tests were normal, indicating the absence of infection. No significant differences were identified between genders (Table 2).

No patients had reported or developed tea-colored urine, and no significant abnormalities were seen in available routine urinalysis of most patients, while five out of 30 patients (with routine urinalysis results) had elevated specific gravity (SG) (1.030, normal 1.025); five got positive in urine occult blood test (graded as 1+ to 3+), and available microscopic analysis on majority of the patients showed few, if any, red blood cells, except the patient with 3 ± positive to hemoglobin (26 RBC/μl in urinary sediment); and eight present slightly turbid urine color. No sustained decrease of urine output was reported.

Treatment consisted of intravenous hydration, with administration of hepatic and stomach protective agents as well as diuretics. The drugs administered included glucurolactone, pantoprazole, furosemide, bicarbonate, reduced glutathione, inosine, cimetidine, calcium gluconate, and potassium chloride.

### Risk factors and source of disease

None of the patients had recently travelled out of town, and none had related medical histories such as trauma, underlying cardiovascular and/or brain diseases, medications. The only common factor was exposure to Pomfrets bought from local markets on 26 October, 2009. In all cases the fish were cooked thoroughly without heads or internal organs. There were no reports that the fish had an unusual odor or taste. The patients consumed the Pomfrets for supper on 26 October, or for breakfast on 27 October, by themselves or with family members or workmates. The estimated weight of fish eaten ranged from 50 to 700 g and was related to the severity of muscle pain. Based on the time to disease onset relative to the consumption of the suspect meals, the median incubation period of the disease was estimated to be 7 h (range: 10 min to 41.5 h).

Tracing investigations, indicated that the suspect Pomfrets had been cultivated with Silver carp *(Hypophthalmichthys molitrix)*, Grass carp *(Ctenopharyngodon idella)*, and bighead carp *(Aristichthys nobilis)*, in a reservoir named ‘Lengshuidong’. Though it was the correct season, the Pomfrets caught from the reservoir were much smaller (mostly < 200 g) than the expected average weight (> 1 kg), and were also smaller than Pomfrets from the same batch of fish fry that had been cultivated elsewhere.

As these fish were too small to be sold at a favorable price, the wholesalers purchased other kinds of fish from the reservoir for resale in Lianzhou. No disease was reported after consumption of these other species. The low weight Pomfrets were sold at very low prices to the residents of three villages of Jiubei. The four patients from Liannan County had bought and consumed Pomfrets from the same lot.

**Figure 2:**
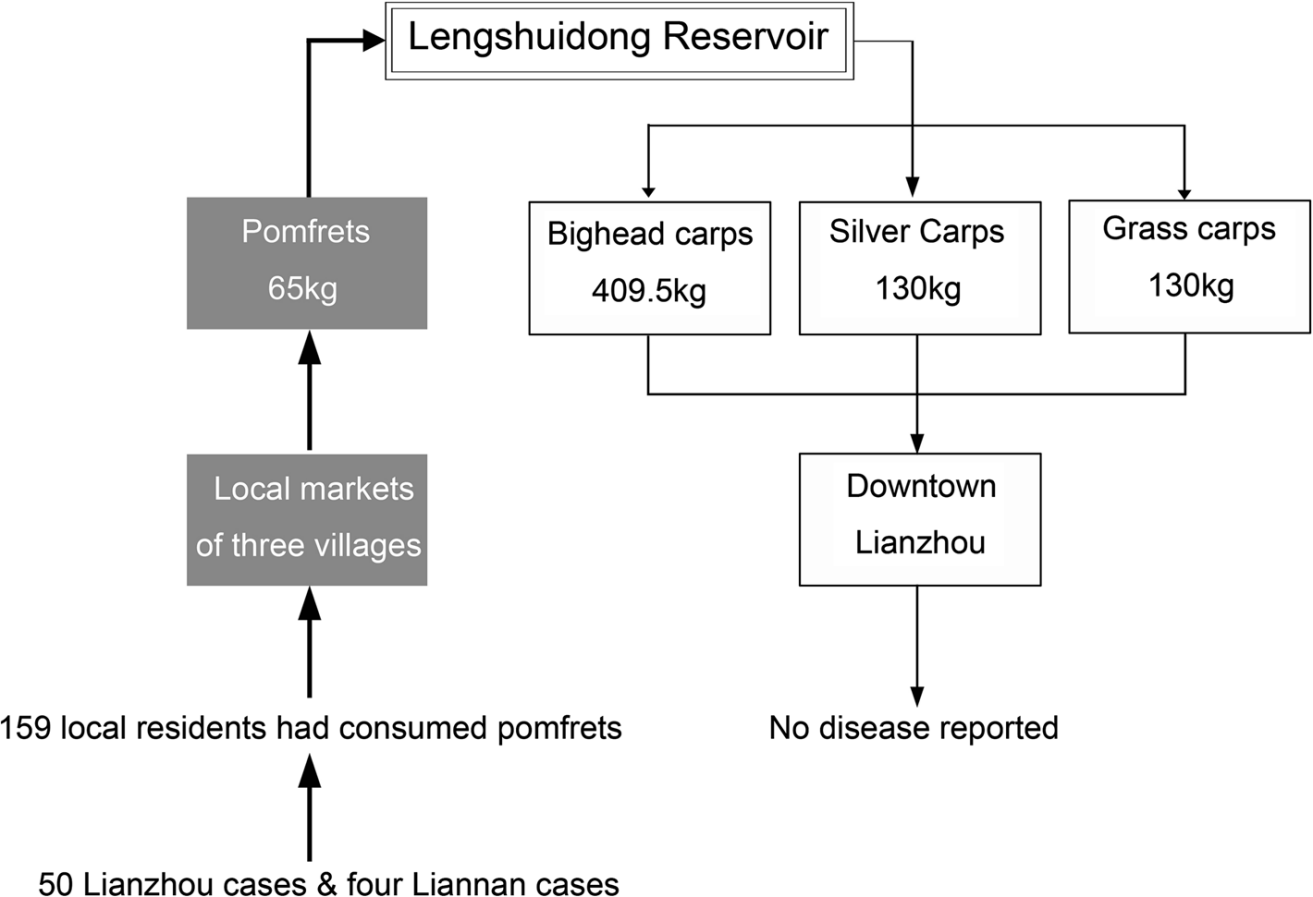
Network graphic on the tracing investigating of fish caught from Lengshuidong reservoir, in outbreak occurred in town of lianzhou, Guangdong, South China, 2009.

Figure 2 shows the results of tracing investigations for Pomfrets and other fish cultivated at the same reservoir. The reservoir for fish aquaculture covered about 26.67 hectares and was at least three meters deep during the wet season, being supplied by water coming from the surrounding mountains. No industrial or mining contamination was possible, but there was a sty with 400 pigs at the bank of the reservoir. This is a typical method of cultivation in rural areas of China, with the feces and waste products of the livestock discharging into the reservoir as fish food. The reservoir had been used to cultivate Pomfret and other kinds of fish since 2007. No unexpected fish or animal deaths had been reported in the reservoir area.

### Oral toxicity testing

Five groups of mice (A to E) were subjected to different fish samples. Groups A and B were subjected to Pomfrets from Lengshuidong reservoir, Group C were subjected to patients’ leftovers and Groups D and E were respectively subjected to bighead carp from the same reservoir and to Pomfrets cultivated in a nearby pond. Another five groups of mice (F to J) were subjected to water samples collected from surface (F), bottom (G) and sewage draining outlet (H) of the Lengshuidong reservoir. Groups I and J were subjected to water collected from another reservoir.

Two hours after administration, mice in Groups A and C showed evidence of deteriorating mental states; they became less active, and developed akinesia in the hind limbs, causing them to lie down or bend over. Three mice died after 22 h (two in Group A and one in Group C), but had no obvious abnormalities in gross anatomy. NO significant abnormalities were seen in mice from any of the other groups.

### Chemical analyses

Chemical analyses of Pomfret samples collected from Lengshuidong reservoir or from patients’ leftovers, together with water and soil samples on possible toxins and metals were either negative or below toxicity thresholds.

### Study of the specific risk factors

A retrospective cohort study on 3,857 Jiubei residents revealed that 159 subjects had consumed Pomfrets on 26 October 2009, of whom 50 (excluding the four Liannan cases) fell ill. This resulted in an attack rate of 31.5% (50/159). None of the patients who did not consume Pomfrets presented with similar symptoms (*P* < 0.001 Fisher exact test). Thus, neither the relative risk nor 95% confidence interval could be calculated.

A dose response relationship was demonstrated between the weight of Pomfret consumed and elevated enzymes levels including CK-MB (*R*^*2*^ = 0.713, *P* < 0.001), and AST (*R*^*2*^ = 0.387, *P* = 0.019), CK (*R*^*2*^ = 0.319, *P* = 0.049) by fitting cubic model. Higher weights of fish consumption were associated with higher enzyme activity and more severe symptoms.

### Control measures

On the afternoon of 27 October 2009, local government and public health departments issued a warrant prohibiting fishing, selling and consumption of Pomfrets from Lengshuidong reservoir. They also alerted hospitals to search their medical records as soon as possible to identify patients who had consumed Pomfrets and felt uncomfortable afterwards. These procedures ensured that no new case emerged after 2 November 2009 (Figure 1).

## Discussion

After being initially identified in Europe in 1924, a second Haff disease epidemic was reported in the same region in 1932. Similar epidemics and/or sporadic cases were reported in Sweden, Russia. In the United States, there were two cases a year reported between 1984 and 1986. In 1997 there were six cases, in 2009 there were nine cases and in 2011 there were three cases. In China, six cases of the disease were reported in 2000, an outbreak consisted of 23 cases was occurred in 2010, in Brazil 25 cases were reported in 2008 and in Japan 13 cases were reported in 2009 [9-16].

Typical clinical features of the condition comprise severe generalized muscle pain and tenderness with weakness, 5-times or greater elevation in levels of serum CK, with a CK muscle/brain (MB) fraction < 5% [6]. No accompanying fever, neurological, hepatic and/or splenic abnormalities are observed. Most patients recover without sequelae, and very few die. Recent ingestion of cooked fishery product is the culprit in most cases, with incubation periods generally ranging from 6 to 8 h [5,9,12]. Reported fishery products include buffalo fish *(Ictiobus cyprinellus)*, burbot, eel, pike, crayfish, salmon, silver dollars (*Mylossoma* spp.), black-finned colossoma (*Colossoma macropomum*), freshwater pompano (*Piaractus brachypomus*), and marine box fish, all of which are predominantly bottom-feeding omnivors [3,5,9,13,16].

The absence of fever and the fact that fishery products were cooked thoroughly excludes the possibility of known infectious causes. Proposals that the etiologies may include arsenic [5] or palytoxin [9] remain unconfirmed [5,10].

Thorough investigation into previous Haff disease epidemics indicated that the 2009 outbreak in South China was not simply an incident of acute muscle poisoning but was an outbreak of Haff disease itself. This conclusion was based on the clinical characteristics, which included typical symptoms and clinical laboratory findings associated with rhabdomyolysis. All affected patients reported consumption of self-prepared Pomfrets in the 24 h before the onset of symptoms. Some of the cases occurred in clusters, and the majority had a sudden onset with a median incubation period of only 7 h. Subjects from the same region who had not consumed Pomfrets that day did not present with similar symptoms. Experimental studies indicated that the Pomfrets not only caused human illness, but also intoxicated laboratory mice. Pomfret, in common with other fishery products that have been reported to cause Haff disease, is a bottom-feeder that has the potential to accumulate environmental toxins.

## Conclusions

This study identified a previously misdiagnosed and neglected outbreak of Haff disease which comprised the highest number of reported cases to date in China or Asia. Analysis of this outbreak indicated that Haff disease can migrate, as it has already traveled from Haff Shore in the Baltic to Asia. The disease not only occurs in sporadic cases or clusters, but also in relatively large-scale outbreaks. It is spread by wild fishery products, as well as by fish farmed from aquaculture centres. The toxin(s) responsible for Haff disease is fishery product borne, heat-stable and cannot be identified by smell or taste [5,7]. The outbreak also highlights the risks of recurring food borne diseases, such as Haff disease, to the public, as increasing numbers of health-conscious subjects prefer fish diets.

The potential for misdiagnosing Haff disease is considerable, and the toxin itself has yet to be identified. In the meantime there is a clear need for sensitive, ongoing surveillance procedures as well as precise diagnostic methods, and effective precautionary procedures. Our retrospective analysis indicated that, in addition to a limited knowledge base on unusual diseases, separation between epidemiological and clinical investigations led to passive actions being taken. High dependency on laboratory results continues to be the norm in food borne outbreak investigations in China and this alone may hinder accurate diagnosis.

## Limitation of the study

Since our study was based on past time investigations and comparisons, some limitations cannot be ignored: first, separation between epidemiological and clinical investigations led to challenging of the original diagnosis and clinical analyses during outbreak impossible, and also resulted in missing of some important clinical data. Second, the retrospective cohort study was limited for methodological reasons to the exposure, the freshwater Pomfret, that were able to explain a large population of cases, but neglected other variables which might possibly be significant. Third, limitation of knowledge on Haff disease and insufficiency of fish samples during outbreak made the animal tests were only used to ascertain that the suspected Pomfrets were toxic, and no further analyses on toxic animals were done to identify the consistency with toxic patients.

## Abbreviations

CK: Creatine kinase; CDC: Centre for disease control and prevention; MTD: Maximal tolerate dose; α-HBDH: α-hydroxy butyrate dehydrogenase; AST: Aspartate aminotransferase; LDH: Lactic dehydrogenase; TBA: Total bile acid; ALT: Alanine aminotransferase; Cr: Creatinine; BUN: Blood urine nitrogen; SG: Specific gravity; LC-MS: Liquid chromatography-mass spectrometry; IC: Iron chromatography; GC-MS: Gas chromatography–mass spectrometry; ICP-MS: Inductively coupled plasma mass spectrometry; AFS: Atomic fluorescence spectrometry

## Competing interests

The authors declare that they have no competing interests.

## Authors’ contributions

XH, YPL, QH, JHL, YHZ, led the outbreak investigation; CSL, BFC implemented the sampling and toxicity tests, and chemical analyses, respectively. XH, QH, JHL, XLD,YHZ, YNW, BS conceived further studies. XH, YPL, QH, JHL, LLL, ZHC led all aspects of data collection, literature reviews, statistical analysis. XH, YPL, JHL developed the draft manuscript under directions of YHZ & QH; XH finished the submitted version and took charge of the whole submission and revision process. All authors participated in the analysis and interpretation of the results, as well as read and approved the final edition and the revised version of the manuscript.

## Pre-publication history

The pre-publication history for this paper can be accessed here:

http://www.biomedcentral.com/1471-2458/13/447/prepub
